# Concurrent Cholecystectomy Is Associated with a Lower Risk of Recurrence after Curative Resection in Early-Stage Hepatocellular Carcinoma: A 10 Year Observational Single-Center Study

**DOI:** 10.3390/jpm11121261

**Published:** 2021-11-30

**Authors:** Yu-Syuan Chen, Shih-Yu Yang, Pei-Ming Wang, Chih-Chi Wang, Chee-Chien Yong, Ding-Wei Chen, Yueh-Wei Liu, Ching-Hui Chuang, Pao-Yuan Huang, Chih-Chien Yao, Yen-Po Lin, Ming-Chao Tsai

**Affiliations:** 1 School of Medicine, Chung-Shan Medical University, Taichung 40201, Taiwan; dante44442@gmail.com (Y.-S.C.); youcryforlol@gmail.com (Y.-P.L.); 2 Division of Hepato-Gastroenterology, Department of Internal Medicine, Kaohsiung Chang Gung Memorial Hospital, Chang Gung University College of Medicine, Kaohsiung 83301, Taiwan; three0310@gmail.com (S.-Y.Y.); paoyuan813@gmail.com (P.-Y.H.); chihchienyao@gmail.com (C.-C.Y.); 3 Department of Family Medicine, Kaohsiung Chang Gung Memorial Hospital, Kaohsiung 83301, Taiwan; wangpeming@yahoo.com.tw; 4 Liver Transplantation Center and Department of Surgery, Kaohsiung Chang Gung Memorial Hospital, Chang Gung University College of Medicine, Kaohsiung 83301, Taiwan; ufel4996@gmail.com (C.-C.W.); yong3980@hotmail.com (C.-C.Y.); Anthony0612@me.com (Y.-W.L.); 5 Center for Translational Research in Biomedical Sciences, Liver Transplantation Program and Department of Surgery, Kaohsiung Chang Gung Memorial Hospital, Chang Gung University College of Medicine, Kaohsiung 83301, Taiwan; dennis8870@gmail.com; 6 Department of Nursing, Meiho University, Pingtung 91202, Taiwan; Helen.ch.chuang@gmail.com; 7 Graduate Institute of Clinical Medical Sciences, College of Medicine, Chang Gung University, Taoyuan 33302, Taiwan

**Keywords:** cholecystectomy, hepatocellular carcinoma, recurrence

## Abstract

Background: Cholecystectomy has been reported to be associated with increased risk of developing hepatocellular carcinoma (HCC). However, there is little information about the impact of cholecystectomy on the outcome of HCC. Aims: To evaluate the long-term effect of concurrent cholecystectomy on recurrence and overall survival in HCC after curative hepatectomy. Patients and Methods: We retrospectively enrolled 857 patients with BCLC stage 0 or A HCC who underwent primary resection from January 2001 to June 2016. The impact of concurrent cholecystectomy on overall survival (OS) and recurrence-free survival (RFS) were analyzed by Cox’s proportional hazards models after one-to-one propensity score matching (PSM). Results: Of the 857 patients, 539 (62.9%) received concurrent cholecystectomy (cholecystectomy group) and 318 (37.1%) did not (non-cholecystectomy group). During the mean follow-up period of 75.0 months, 471 (55.0%) patients experienced recurrence, and 321 (37.5%) died. RFS and OS were not significantly different between the groups. After PSM, a total of 298 patients were enrolled in each group. RFS was significantly higher in the cholecystectomy than non-cholecystectomy group (*p* = 0.044). In multivariate analysis, age (*p* = 0.022), serum AFP (*p* = 0.008), liver cirrhosis (*p <* 0.001), diabetes (*p* = 0.004), tumor number (*p* = 0.005), tumor size (*p* = 0.002), histological grade (*p* = 0.001), microvascular invasion (*p* < 0.001) and cholecystectomy (*p* = 0.021) were independent risk factors for HCC recurrence. However, there were no significant differences in OS between the cholecystectomy and non-cholecystectomy groups. Conclusions: Concurrent cholecystectomy may reduce recurrence in early-stage HCC after curative resection. Further studies are needed to validate our results.

## 1. Introduction

Hepatocellular carcinoma (HCC), the most common primary malignancy of the liver, is the second leading cause of cancer-related deaths in the world [1]. The major risk factors for HCC are hepatitis B virus (HBV), hepatitis C virus (HCV), aflatoxin, excessive alcohol consumption, obesity and diabetes mellitus (DM) [2]. Current management strategies for HCC depend on the tumor stage and include surgical resection, liver transplantation, radiofrequency ablation (RFA), transarterial chemoembolization, radiation therapy and systemic therapy [3]. To date, surgical resection is widely accepted as an effective treatment for patients with early-stage HCC who have well-preserved liver function. Nevertheless, the overall survival after curative resection remains unsatisfactory because of the high rate of recurrence. Around 50–70% of patients with HCC who undergo potentially curative surgical resection develop recurrence within 5 years [4,5,6]. Many factors are known to affect the risk of recurrence in HCC, including tumor size, alpha-fetoprotein (AFP), microvascular invasion, cirrhosis, resection margin, and the viral replication status of HBV and HCV [6,7,8,9,10].

Cholecystectomy is the preferred treatment option for gallbladder (GB) diseases [11]. Recently, an epidemiological investigation and meta-analysis showed that cholecystectomy was associated with an increased risk of developing HCC [12]. The majority of related studies indicate that cholecystectomy is associated with an increased risk of developing HCC. A Korean study suggested that increased exposure of the digestive tract to bile, changes in metabolic hormone levels, or long-lasting inflammation after cholecystectomy are possible biological mechanisms that may contribute to the increased incidence of various types of cancer in patients who have undergone cholecystectomy [13].

Although a previous study reported cholecystectomy was associated with a higher risk of early recurrence and poorer survival after curative resection for early-stage HCC [14], there were still many issues unresolved. The most important was that the surgical indication of concurrent cholecystectomy in HCC patients is not widely advocated. There is a paucity of studies on the impact of concurrent cholecystectomy in cases of HCC after resection. In this study, we retrospectively analyzed data on 857 patients with Barcelona Clinic Liver Cancer (BCLC) stage 0 or A HCC who underwent primary curative resection to investigate the impact of cholecystectomy on postoperative recurrence and survival in HCC.

## 2. Materials and Methods

### 2.1. Study Design

**Figure 1:**
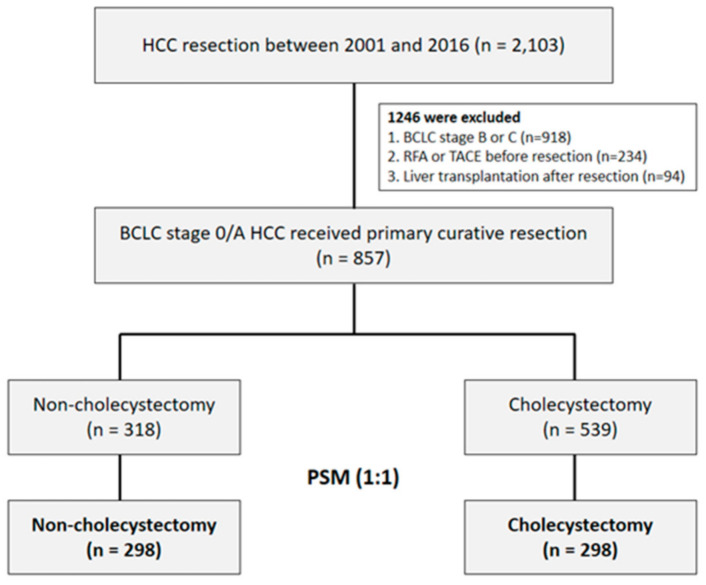
Patient selection flow diagram.

The data used in this retrospective study were extracted from our hospital HCC registry database. A total of 2103 patients diagnosed with HCC who underwent surgical resection between January 2001 and June 2016 were retrospectively enrolled. We excluded 918 patients with Barcelona Clinic Liver Cancer (BCLC) stage B or C HCC, and 234 patients who underwent treatment prior to surgery. In well-selected patients, liver transplantation is generally considered to cure the tumor and underlying cirrhosis at the same time, and thus strongly influences survival and recurrence [15]. Therefore, 94 patients who underwent salvage liver transplantation were also excluded. Finally, a total of 857 patients with BCLC stage 0 or A HCC who underwent primary curative resection (Figure 1) were included in this study.

This study was performed in compliance with the standards of the Declaration of Helsinki and current ethical guidelines, and is reported in line with the STROCSS criteria [16]. The clinical data were acquired with the approval and permission of the Institutional Review Board of the Kaohsiung Chang Gung Memorial Hospital (approval number: 201901103B0). The study protocol was approved by the Institutional Review Board of Kaohsiung Chang Gung Memorial Hospital. Informed consent was not required because this study was a retrospective analysis of clinical data, with no relevant human biological or ethical issues. Written informed consent was obtained from all patients for use of their data for research purposes.

### 2.2. Study Assessments and Follow-Up Evaluation

The medical records were reviewed to obtain data on patient demographics and clinical characteristics, including serum biochemistry, albumin and alpha-fetoprotein (AFP), Child-Pugh classification, viral hepatitis status, duration of follow-up and outcomes. A comprehensive range of tumor characteristics, including satellite nodules, capsule invasion, microvascular invasion and tumor differentiation, were carefully recorded from the electronic medical records for analysis. The presence or absence of gallbladder (GB) stones and cholecystectomy/no-cholecystectomy were also recorded. In addition, the type of surgery, i.e., segmentectomy or lobectomy, and open surgery or laparoscopic surgery, was recorded.

The patients were followed up 1 month after surgery, every 3 months in the first year, and every 3–6 months in subsequent years. Serum AFP and serum biochemistry were assessed and abdominal ultrasonography was performed at every follow-up. Dynamic computed tomography or magnetic resonance studies were performed 1 month after resection and every 12 months thereafter, or if HCC recurrence was clinically suspected. The last follow-up was 30 April 2020. Recurrence-free survival (RFS) was defined as the interval between surgery and the date of diagnosis of the first recurrence; overall survival (OS) was defined as the interval between surgery and death or last follow-up.

### 2.3. Definitions

All the diagnoses of HCC were confirmed by contrast-enhanced multiphase computed tomography (CT) or magnetic resonance imaging (MRI), in accordance with the criteria of the practice guidelines of the European Association for the Study of the Liver (EASL) and the American Association for the Study of Liver Disease (AASLD) [17,18], and were pathologically confirmed after surgical resection. The BCLC system is endorsed by the EASL [19] and AASLD [20], and the revision of a single large HCC (>5 cm) to BCLC stage B (instead of stage A) was widely adopted by the Taiwan Liver Cancer Association (http://www.tlcaweb.org.tw/ accessed on 30 April 2020). The histologic grade of tumor differentiation was scored using the modified nuclear grading scheme outlined by Edmondson and Steiner, and categorized as well, moderately, or poorly differentiated [21]. For patients with multifocal disease, the tumor grade was coded as the highest grade of any of the tumors. The degree of hepatic fibrosis was scored in surgically resected non-tumor tissues according to the classification of Ishak et al. [22]. Liver cirrhosis was defined as an Ishak fibrosis score of 5–6.

### 2.4. Statistical Analysis

Propensity score matching (PSM) was applied to reduce selection bias between the study groups. Age, sex, diabetes mellitus, hepatitis B, hepatitis C, serum AST, serum ALT, platelets, total bilirubin, serum albumin, liver cirrhosis, Child–Pugh grade, BCLC stage, serum AFP, tumor size, tumor number, vascular invasion, histology, hepatectomy type and cholecystectomy (yes/no) were selected as independent variables. The Greedy method of NCSS 10 Statistical Software (NCSS LLC, Kaysville, UT, USA) was used to match the study groups in a 1:1 ratio; the caliper width was 0.2 of the standard deviation of the propensity score between the study groups. The standardized mean difference (SMD) was used to evaluate the balance of covariates after PSM.

The demographic data were compared between the groups using Fisher’s exact test or the chi-square test, as appropriate. Continuous variables are expressed as the median ± interquartile range (IQR). A time-dependent, receiver-operating characteristic (ROC) curve analysis was used to assess the optimal cut-off points for age, tumor size, and serum AFP using Youden’s index for the prediction of tumor recurrence and overall survival [23]. The Kaplan–Meier method was used to plot the RFS and OS curves stratified by cholecystectomy; the curves were compared using the log-rank test. Factors that were significant in the univariate analysis (*p* < 0.05) were included in the multivariate analyses of OS and RFS using a Cox forward stepwise variable selection process. Hazard ratios (HR) and 95% confidence intervals (CI) were also calculated for each factor. Statistical analyses were performed using SPSS 22.0 software (SPSS Inc., Chicago, IL, USA). All the statistical tests were two-sided; the *p*-values < 0.05 were considered significant.

## 3. Results

### 3.1. Patient Characteristics

The characteristics of the cohorts of unmatched and matched patients are presented in Table 1. The mean follow-up time was 82.7 months (the range: 1–230 months) after liver surgery. The unmatched patient cohort included 670 men and 187 women, with a median age of 59 years at enrollment. Overall, 222/857 patients (25.9%) had diabetes before surgery and 400 patients (46.7%) were diagnosed with cirrhosis. Of the 857 patients, 539 (62.9%) received cholecystectomy (cholecystectomy group) and 318 (37.1%) did not (non-cholecystectomy group). Compared to the non-cholecystectomy group, the patients in the cholecystectomy group had higher serum albumin (*p* = 0.041), a higher frequency of Child–Pugh grade B HCC (*p* = 0.008), larger tumors (*p* = 0.001), a higher number of tumors (*p* =   0.018), and more frequently underwent liver lobectomy (*p* < 0.001) and open cholecystectomy (*p* < 0.001). Overall, recurrence and death were not significantly different between the cholecystectomy group and the non-cholecystectomy group before propensity score matching (*p* = 0.684 and *p* = 0.518, respectively).

After 1:1 PSM, 298 patients in the cholecystectomy group and 298 patients in the non-cholecystectomy group were analyzed. All the baseline characteristics were balanced between the matched groups (SMD < 0.2 and *p* >  0.05 for all variables; Table 1).

### 3.2. Survival Analysis before and after Propensity Score Matching Analysis

**Figure 2:**
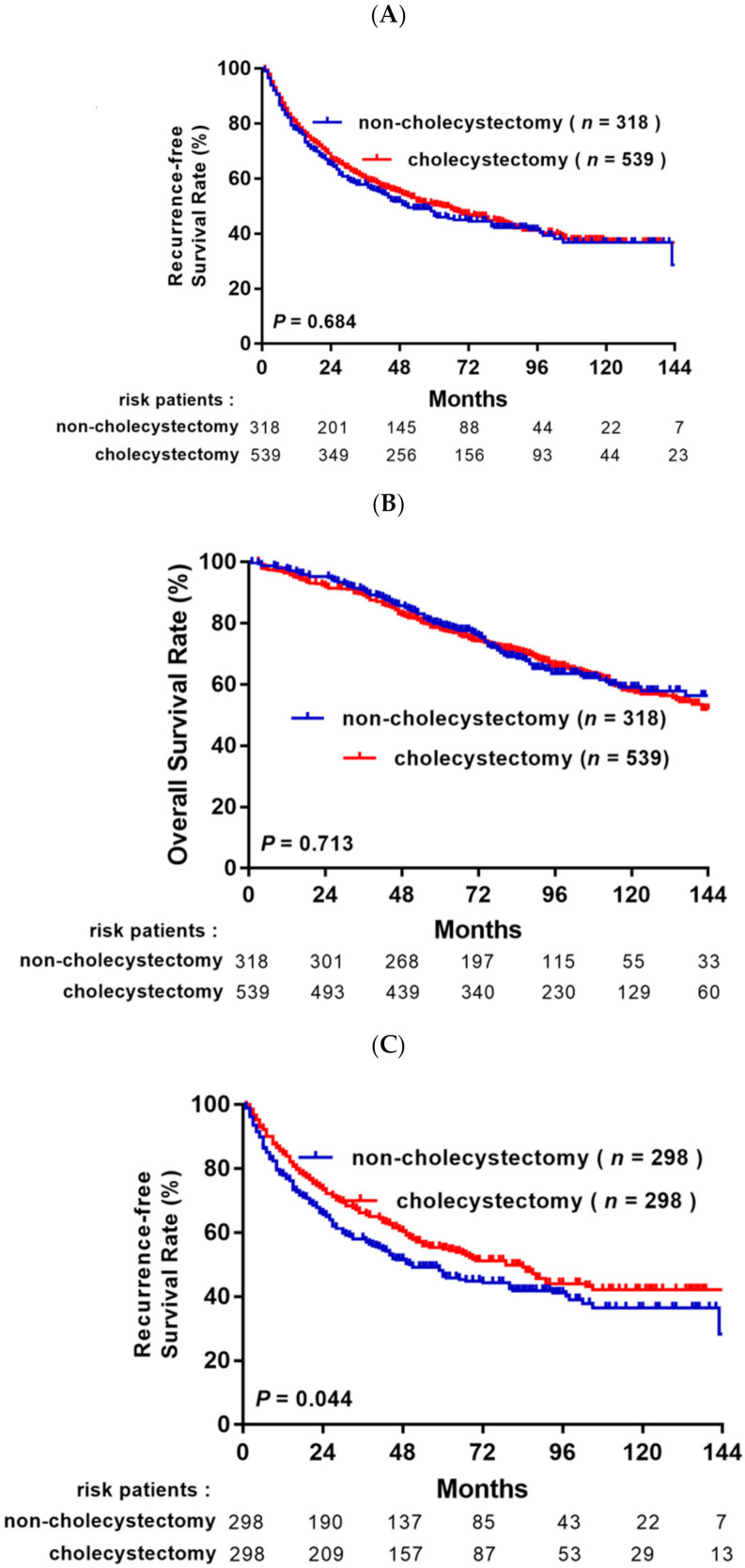

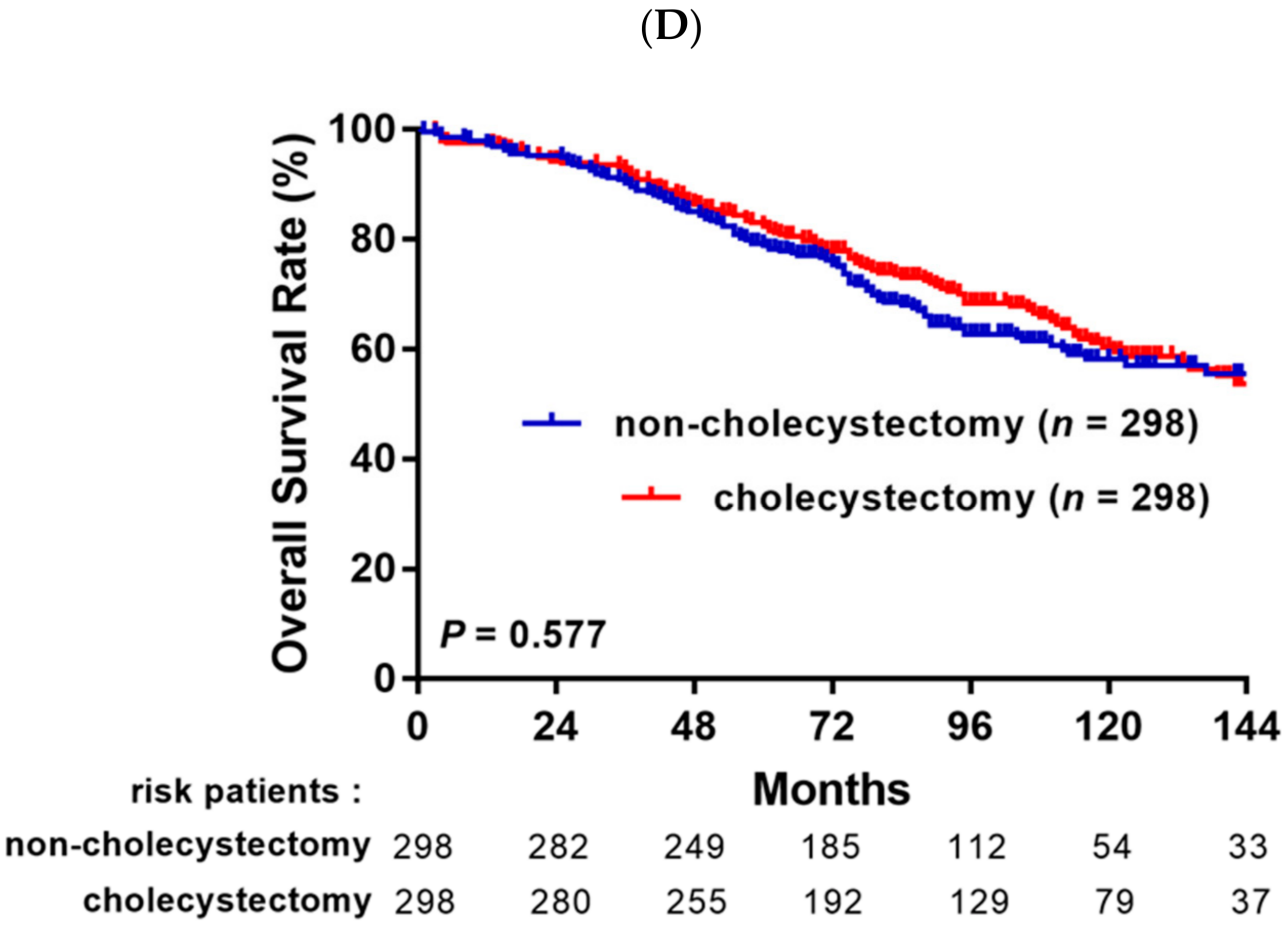
Recurrence-free survival (RFS) and overall survival (OS) after resection for HCC patients with or without cholecystectomy before propensity score matching (PSM) (**A**,**B**) and after PSM (**C**,**D**).

In the unmatched cohort, a total of 471/857 (55%) patients developed recurrence during the mean follow-up period of 75.0 months. The 1, 3, 5, and 10 year RFS rates were 77.7%, 57.9%, 48.2% and 36.8% and 79.2%, 59.5%, 49.9% and 36.4% in the non-cholecystectomy group and the cholecystectomy group, respectively (*p* = 0.684, Figure 2A). In the OS analysis, a total of 321 (37.5%) patients died during follow-up. The 1, 3, 5, and 10 year OS rates were 97.5%, 90.8%, 79.6%, and 58.9% and 96.5%, 89.5%, 78.1%, and 57.6% in the non-cholecystectomy group and the cholecystectomy group, respectively (*p* = 0.518, Figure 2B).

After PSM, the 1, 3, 5, and 10 year RFS rates were 77.9%, 58.1%, 48.2% and 36.4% and 84.3%, 66.0%, 54.7% and 42.0% in the non-cholecystectomy group and the cholecystectomy group, respectively (*p* = 0.044, Figure 2C); however, the 1, 3, 5, and 10 year OS rates were 97.3%, 90.5%, 78.9% and 58.0% and 97.6%, 93.3%, 82.7% and 60.2% in the non-cholecystectomy group and the cholecystectomy group, respectively, with no significant difference in OS between the two matched groups (*p* = 0.518, Figure 2B).

**Figure 3:**
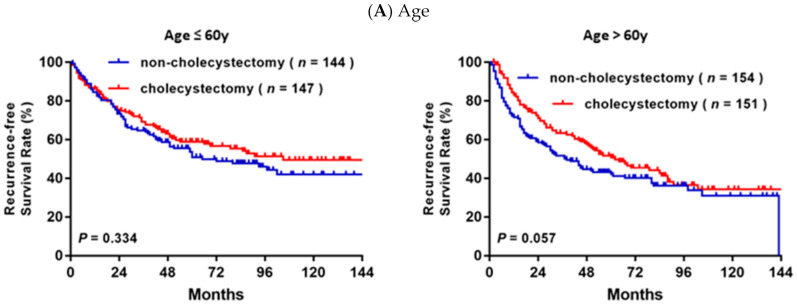

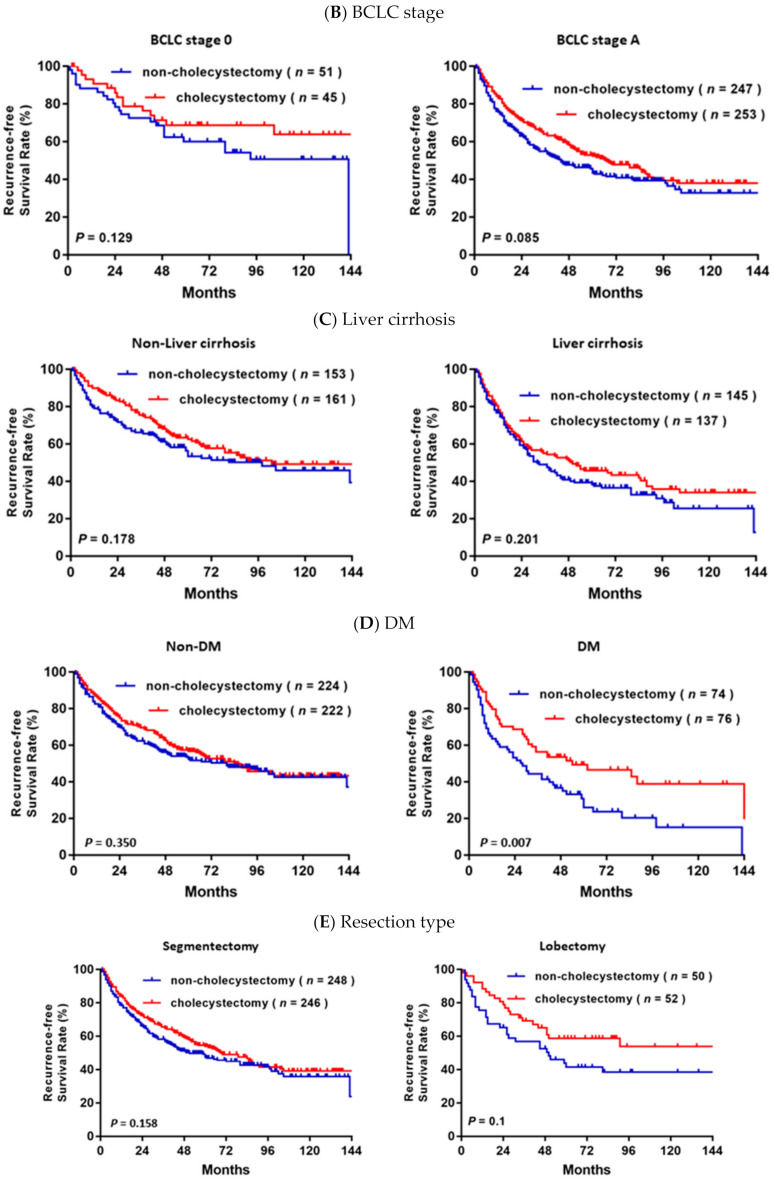

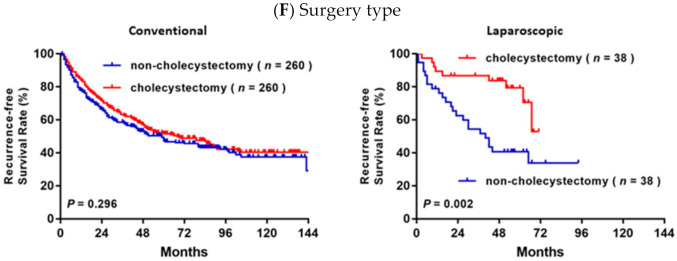
Kaplan-Meier cumulative recurrence-free survival after resection for HCC patients with or without cholecystectomy stratified by (**A**) age, (**B**) BCLC stage, (**C**) liver cirrhosis, (**D**) diabetes, (**E**) resection type and (**F**) surgery type.

In the subgroup analysis based on various clinical characteristics (Figure 3), RFS was significantly higher in the cholecystectomy group than the non-cholecystectomy group in the subgroups of patients with DM (*p* = 0.007, Figure 3D) and who underwent laparoscopic cholecystectomy (*p* = 0.002, Figure 3F). Although there were no significant differences in RFS and OS in the subgroups stratified by age, BCLC stage, cirrhosis, or resection type, a trend towards better RFS was observed in the cholecystectomy group compared to the non-cholecystectomy group.

### 3.3. Univariate and Multivariate Analyses of Independent Risk Factors

Univariate analyses demonstrated that serum AFP, liver cirrhosis, DM, Child–Pugh grade, BCLC stage, tumor number, tumor size, histological grade, vascular invasion and cholecystectomy were significantly associated with RFS (Table 2). In the multivariate analysis, older age (HR, 1.305; 95% CI, 1.039–1.638; *p* = 0.022), serum AFP > 5 ng/mL (HR, 1.443; 95% CI, 1.101–1.890; *p*  = 0.008), liver cirrhosis (HR, 1.541; 95% CI, 1.227–1.935; *p* < 0.001), DM (HR, 1.433; 95% CI, 1.118–1.836; *p* = 0.004), multiple tumors (HR, 1.861; 95% CI, 1.211–2.859; *p*  = 0.005), tumor size >2 cm (HR, 1.501; 95% CI, 1.154–1.951; *p*  = 0.002), poorly differentiated tumors (HR, 2.411; 95% CI, 1.422–4.085; *p* = 0.001), vascular invasion (HR, 1.505; 95% CI, 1.196–1.893; *p* < 0.001) and cholecystectomy (HR, 0.770; 95% CI, 0.616–0.962; *p* = 0.021) remained independent prognostic factors for RFS.

In the OS analysis, the multivariate Cox proportional hazards model revealed that serum AFP > 5 ng/mL (HR, 1.592; 95% CI, 1.135–2.232; *p*  = 0.007), liver cirrhosis (HR, 2.019; 95% CI, 1.527–2.670; *p* < 0.001), DM (HR, 2.214; 95% CI, 1.675–2.927; *p* < 0.001), Child–Pugh grade B (HR, 2.310; 95% CI, 1.431–3.731; *p* = 0.001), multiple tumors (HR, 1.679; 95% CI, 1.029–2.740; *p* = 0.038), tumor size >2 cm (HR, 1.523; 95% CI, 1.109–2.094; *p* = 0.009) and vascular invasion (HR, 1.498; 95% CI, 1.129–1.986; *p*  = 0.005) were independent risk factors associated with OS (Table 3).

### 3.4. Prognostic Value of Cholecystectomy Based on Gallbladder Stones

**Figure 4:**
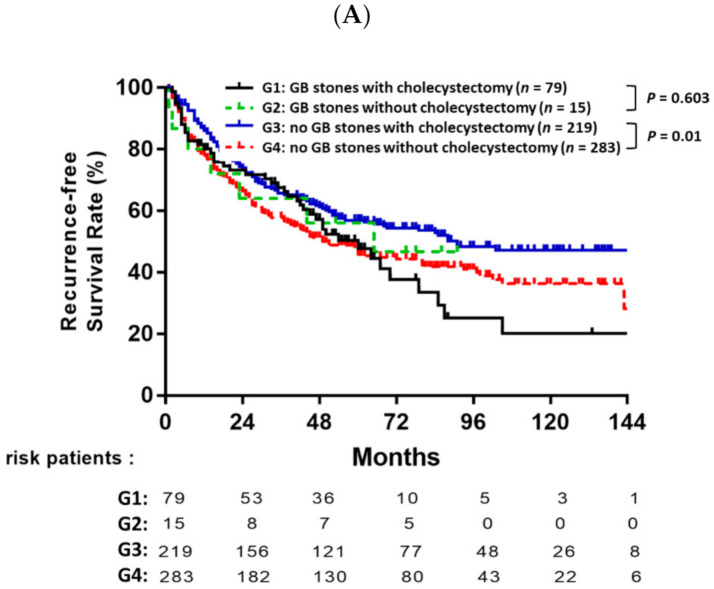

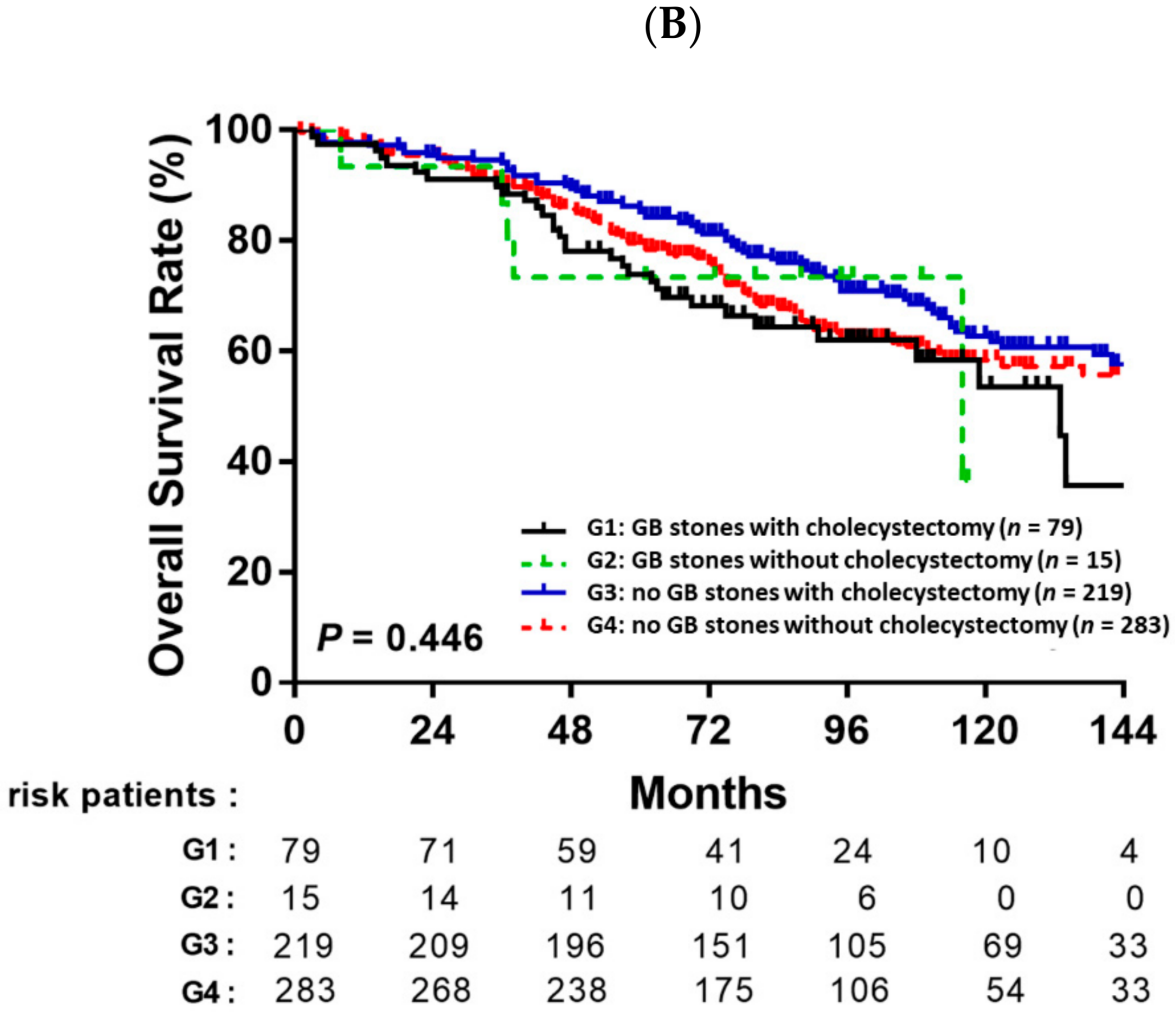
Recurrence-free survival (**A**) and overall survival (**B**) after resection for HCC patients stratified by the presence or absence of gallbladder stones and cholecystectomy or no cholecystectomy.

Overall, the entire cohort of 857 patients was divided into four subgroups: GB stones with cholecystectomy (*n* = 79), GB stones without cholecystectomy (*n* = 15), no GB stones with cholecystectomy (*n* = 219) and no GB stones without cholecystectomy (*n* = 283). Among the patients without GB stones, patients receiving cholecystectomy had significantly better RFS rates than the patients that did not undergo cholecystectomy (*p* = 0.01, Figure 4A). However, there was no significant difference in OS between patients who underwent cholecystectomy with GB stones and patients who underwent cholecystectomy without GB stones (Figure 4B).

## 4. Discussion

Liver resection remains the most effective treatment for patients with HCC, but the long-term prognosis after hepatectomy for HCC is still unsatisfactory due to the high rate of intrahepatic recurrence [1,24]. Previous studies showed cholecystectomy was associated with an increased risk of developing HCC [12,13,25], but whether cholecystectomy influences the outcome of patients with HCC after resection was unclear. In this study, focusing on patients with BCLC stage 0 or A HCC, we found that cholecystectomy was associated with a lower rate of recurrence after curative resection in early-stage HCC. To the best of our knowledge, this is the first study to indicate that concurrent cholecystectomy lowers the risk of recurrence in patients with HCC with or without gallstones.

Cholecystectomy is considered the treatment of choice for symptomatic gallstones and gallbladder diseases. However, there is a contradiction between gallbladder stones and cholecystectomy. Gallstones are considered a risk factor for biliary tract cancer, including gallbladder cancer, bile duct cancer and cancer of the ampulla of Vater [26,27]. The relative risks between gallstones and gallbladder cancer ranges from 2.3 to 34.4 in case-control studies [28]. Among patients with gallbladder cancer, 70–90% have a history of cholelithiasis [26,29]. The development of cholelithiasis-related gallbladder cancer may be induced and/or promoted by chronic irritation and the local production of carcinogens, such as secondary bile acids, which lead to epithelial dysplasia and carcinoma. Thus, the concept that cholecystectomy to remove gallbladder stones decreases the risk of gallbladder carcinogenesis is reasonable. Nevertheless, many studies have reported that cholecystectomy is associated with an increased risk of developing HCC [12,25,30]. The pathophysiology of tumorigenesis after cholecystectomy is not well understood. The possible mechanisms are that cholecystectomy is typically followed by the dilation of the common biliary duct and a rise in bile duct pressure, both of which may increase the risk of chronic inflammation and the proliferation of hepatocytes. Chronic inflammation is an accepted carcinogenic mechanism in several types of cancer, including HCC, and chronic inflammation promotes cell proliferation in most liver diseases. Hence, whether cholecystectomy provides a benefit or not is clearly a dilemma. Prophylactic cholecystectomy would appear to be reasonable in selected populations of patients with HCC; however, this surgical indication is not widely advocated and is even controversial.

A recent cohort study by Li et al. demonstrated that concurrent cholecystectomy increased the risk of recurrence and negatively affected survival after surgical resection in early-stage HCC [14]. In contrast, our study indicates that cholecystectomy may reduce the risk of tumor recurrence after curative resection in patients with HCC. This discrepancy may be related to the heterogeneity between these studies. First, in the study by Li et al., the patients in the cholecystectomy group were significantly older than those in the non-cholecystectomy group, whereas the age of the two groups in the present study was well-balanced (59 years, both). Second, Li et al. included patients with single tumors larger than 5 cm, and the mean tumor size was larger in the cholecystectomy group. By contrast, the modified BCLC stage, which excludes large single tumors (>5 cm) from stage A, was adopted in the present study to minimize the tumor burden effect. Third, we applied PSM analysis to minimize selection bias. However, such an analysis was not performed by Li et al. Since no RCTs have been published in this field, our results further emphasize the need for large-scale RCTs to explore the relationship between cholecystectomy and recurrence in HCC.

It is worthy of note that there were 219 patients without GB stones, but receiving cholecystectomy. Because there is a distinct lack of consensus on concurrent cholecystectomy in HCC patients with or without GB stones, the decision to perform cholecystectomy was made by the patients and their clinical physicians. From the baseline data before PSM (Table 1), we found a higher proportion of tumor numbers (10.8% vs. 6.0%, *p* = 0.018), larger tumor size (3.0 ± 1.0 cm vs. 2.7 ± 1.0 cm *p* = 0.001), lobectomy surgery (38.0% vs. 15.7%, *p* < 0.001), and open surgery (91.7% vs. 82.7%, *p* < 0.001) in the cholecystectomy group compared to those in the non-cholecystectomy. Hence, we presumed that the fact that some patients without GB stones received cholecystectomy might have been due to their relatively high tumor burden and the performance of open surgery and lobectomy.

However, we cannot fully explain the mechanisms that underlie the potential effects of cholecystectomy on HCC recurrence observed in the current study. An increasing body of evidence suggests that HCC is associated with changes in the abundance and composition of the intestinal microbiota, as well as impaired barrier function, which induces a leaky gut and gut dysbiosis, and leads to the release of bacteria and their metabolites to the liver. These factors may promote the stepwise progression from fibrosis to cirrhosis and HCC [31]. Furthermore, a recent study from Korea showed that cholecystectomy could alter the gut microbiota [32], possibly due to the increased enterohepatic recirculation of bile acids and increased exposure of the bile acid pool to intestinal bacteria. Although cholecystectomy generally decreases the size of the bile acid pool, the proportion of secondary bile acids with cytotoxic and carcinogenic characteristics increases following the cholecystectomy and, therefore, increases the risk of recurrence in various types of cancers [33]. These established facts were contrary to our results. However, the study by Ma et al. described a mechanism by which the gut microbiome uses bile acids as messengers to control the chemokine-dependent accumulation of hepatic NKT cells and antitumor immunity in the liver, against both primary and metastatic liver tumors [34]. In another study from Taiwan, the Risk Evaluation of Viral Load Elevation and Associated Liver Disease/Cancer (REVEAL) cohort did not support the hypothesis that higher levels of secondary bile acids increase liver cancer risk; in fact, higher levels of the secondary bile acid deoxycholic acid were inversely associated with HCC [35]. This concept is compatible with a recent study from Singapore, in which the ratios of secondary bile acids over primary bile acids were associated with a decreased HCC risk [36]. The results of the present study demonstrate that HCC patients received cholecystectomy with a lower recurrence rate, rather than non-HCC patients receiving cholecystectomy with increasing rates of liver tumors. We think the condition of study population is quite different from those of prior studies. The critical point is that concurrent cholecystectomy might change the balance of microbiota, which has already been established in HCC patients and was associated with HCC development. However, so far, there are no published studies with a similar cohort to ours. A prospective study comparing the change in gut microbiota before and after HCC resection with or without cholecystectomy is needed to prove this result.

Some issues needed to be addressed. After PSM, a total of 315 patients experienced recurrence. Of these, 304 patients (96.5%) experienced intrahepatic recurrence and 11 patients (3.5%) experienced extrahepatic recurrence. There was no significant difference between patients with or without cholecystectomy. Besides, in a total of 596 patients, only one patient underwent R1 resection, which was defined as positive histology margin involvement; the other 595 patients (99.8%) were R0 resection with a resection margin at least 1 cm away from the tumor. The high proportion of R0 section might be explained by the fact that only early-stage HCC patients and those who received curative resection were enrolled in the present study.

In addition to cholecystectomy, we also found that age, liver cirrhosis, serum AFP, diabetes, Child Pugh classification, number of tumors, tumor size, histological grade and microvascular invasion represented independent risk factors for HCC recurrence. These results are consistent with those of previous studies, in which host factors (age and diabetes), liver factors (liver cirrhosis and Child–Pugh classification) and tumor-related factors (serum AFP, tumor size, histological grade and microvascular invasion) were significantly associated with the outcomes of patients with HCC [7,8,24,37,38].

The strength of the present study was the analysis of complete data on a large number of patients with long-term follow-up. We manually reviewed the medical records for each patient, and confirmed their vital status using the Cancers Screening and Tracing Information Integrated System of Taiwan (https://hosplab.hpa.gov.tw/CSTIIS/index.aspx accessed on 30 April 2020). Thus, we could determine the vital status of every single patient enrolled in this study.

There were some limitations to this study. First, this was a retrospective study of patients from a single institution and the data were collected from medical records. Despite employing PSM and multivariable analysis, it was not possible to completely adjust for all the confounding factors. Secondly, this was a single-center study, and there was no universal consensus on concurrent cholecystectomy during HCC resection during the period in which the patients in this study were treated; thus, selection bias may potentially have existed. A validation cohort is necessary to confirm our major findings. Thirdly, we cannot fully explain the mechanisms that underlie the protective effect of concurrent cholecystectomy on HCC recurrence, although we suggest that the effect may possibly be related to changes in the gut microbiota composition after cholecystectomy. Ultimately, further clinical trials that include comprehensive metabolic profiling and analysis of the gut microbiota before and after surgery are required to confirm our findings.

## 5. Conclusions

Concurrent cholecystectomy may reduce recurrence after curative resection in early-stage HCC. Further studies are needed to validate our results.

## Figures and Tables

**Table 1:** Patient Characteristics Before and After Propensity Score Matching. Data are expressed as mean ± standard deviation or n (%). Abbreviations: PSM; propensity score matching; HBV, hepatitis B virus; HCV, hepatitis C virus; AST, aspartate aminotransferase; ALT, alanine aminotransferase; BCLC, Barcelona Clinic Liver Cancer; AFP, alpha fetoprotein; SMD, standardized mean difference.

	Before PSM			After PSM			
Variable	Non-Cholecystectomy (*n* = 318)	Cholecystectomy (*n* = 539)	*p*-Value	Non-Cholecystectomy (*n* = 298)	Cholecystectomy (*n* = 298)	*p*-Value	SMD
Age (years); mean (SD)	58.49 (11.83)	58.71 (11.32)	0.873	58.61 (11.78)	58.69 (10.92)	0.708	0.007
Sex			0.655			0.699	0.032
Male	246 (77.4%)	424 (78.7%)		230 (77.2%)	226 (75.8%)		
Female	72 (22.6%)	115 (21.3%)		68 (22.8%)	72 (24.2%)		
Diabetes	80 (25.2%)	142 (26.3%)	0.701	74 (24.8%)	76 (25.5%)	0.850	0.015
HBV	174 (54.7%)	310 (57.5%)	0.425	166 (55.7%)	161 (54.0%)	0.681	0.034
HCV	110 (34.6%)	190 (35.3%)	0.845	105 (35.2%)	110 (36.9%)	0.670	0.035
AST > 40 U/L	116 (36.5%)	205 (38.0%)	0.649	110 (36.9%)	112 (37.6%)	0.865	0.007
ALT > 40 U/L	135 (41.5%)	239 (44.3%)	0.590	128 (43.0%)	129 (43.3%)	0.934	0.007
Platelets < 150 × 10^3^/µL	155 (48.7%)	256 (47.5%)	0.724	148 (49.7%)	147 (49.3%)	0.935	0.014
Total bilirubin (mg/dL); mean (SD)	0.84 (0.34)	0.81 (0.33)	0.298	0.84 (0.34)	0.83 (0.33)	0.936	0.019
Albumin (g/dL); mean (SD)	3.71 (0.58)	3.60 (0.64)	0.041	3.69 (0.58)	3.68 (0.57)	0.835	0.014
Liver cirrhosis	155 (48.7%)	245 (45.5%)	0.351	145 (48.7%)	137 (46.0%)	0.512	0.054
Child–Pugh grade			0.008			0.708	0.031
A	302 (95.0%)	484 (89.8%)		282 (94.6%)	284 (95.3%)		
B	16 (5.0%)	55 (10.2%)		16 (5.4%)	14 (4.7%)		
BCLC stage			0.160			0.504	0.055
0	53 (16.7%)	71 (13.2%)		51 (17.1%)	45 (15.1%)		
A	265 (83.3%)	468 (86.8%)		247 (82.9%)	253 (84.9%)		
AFP > 200 ng/mL	57 (17.9%)	102 (18.9%)	0.716	53 (17.8%)	53 (17.8%)	1.000	<0.001
Tumor number			0.018			0.480	0.058
Single	299 (94.0%)	481 (89.2%)		279 (93.6%)	283 (95.0%)		
Multiple	19 (6.0%)	58 (10.8%)		19 (6.4%)	15 (5.0%)		
Vascular invasion	113 (35.5%)	207 (38.4%)	0.401	104 (34.9%)	113 (37.9%)	0.444	0.063
Histological grade			0.898			0.932	0.031
Well	40 (12.6%)	71 (13.4%)		38 (12.8%)	35 (11.7%)		
Moderate	269 (84.6%)	446 (84.2%)		251 (84.2%)	254 (85.2%)		
Poor	9 (2.8%)	13 (2.5%)		9 (3.0%)	9 (3.0%)		
Tumor size (cm); mean (SD)	2.7 (1.0)	3.0 (1.0)	0.001	2.8 (1.0)	2.8 (1.0)	0.968	0.006
Resection type			<0.001			0.828	0.018
Segmentectomy	268 (84.3%)	334 (62.0%)		248 (83.2%)	246 (82.6%)		
Lobectomy	50 (15.7%)	205 (38.0%)		50 (16.8%)	52 (17.4%)		
Surgery method			<0.001			1.000	<0.001
Open surgery	263 (82.7%)	494 (91.7%)		260 (87.2%)	260 (87.2%)		
Laparoscopic	55 (17.3%)	45 (8.3%)		38 (12.8%)	38 (12.8%)		

**Table 2:** Univariate and multivariate analysis of recurrence for propensity score-matched patients with BCLC 0/A stage HCC.

Variable	Comparison	Univariate		Multivariate	
		HR (95% CI)	*p*-Value	HR (95% CI)	*p*-Value
Age (years)	>60 vs. ≤60	1.389 (1.111–1.736)	0.004	1.305 (1.039–1.638)	0.022
Sex	Male vs. Female	0.969 (0.745–1.259)	0.811		
AFP (ng/mL)	>5 vs. ≤5	1.495 (1.149–1.944)	0.003	1.443 (1.101–1.890)	0.008
Liver cirrhosis	Yes vs. No	1.676 (1.341–2.093)	<0.001	1.541 (1.227–1.935)	<0.001
Diabetes	Yes vs. No	1.624 (1.274–2.070)	<0.001	1.433 (1.118–1.836)	0.004
Child-Pugh grade	B vs. A	1.359 (0.821–2.251)	0.233		
BCLC stage	A vs. 0	1.701 (1.216–2.381)	0.002		
Tumor number	Multiple vs. Single	1.661 (1.086–2.541)	0.019	1.861 (1.211–2.859)	0.005
Tumor size (cm)	>2 vs. ≤2	1.456 (1.122–1.890)	0.005	1.501 (1.154–1.951)	0.002
Histological grade	Poor vs. well + moderate	2.906 (1.728–4.888)	<0.001	2.411 (1.422–4.085)	0.001
Vascular invasion	Yes vs. No	1.521 (1.211–1.912)	<0.001	1.505 (1.196–1.893)	<0.001
Cholecystectomy	Yes vs. No	0.798 (0.640–0.996)	0.046	0.770 (0.616–0.962)	0.021
Surgery method	Laparoscopic vs. Open surgery	0.838 (0.580–1.210)	0.345		
Resection type	Lobectomy vs. Segmentectomy	0.870 (0.645–1.172)	0.359		

**Table 3:** Univariate and multivariate analysis of overall survival for propensity score-matched patients with BCLC 0/A stage HCC.

Variable	Comparison	Univariate		Multivariate	
		HR (95% CI)	*p*-Value	HR (95% CI)	*p*-Value
Age (years)	>60 vs. ≤60	1.461 (1.114–1.915)	0.006		
Sex	Male vs. Female	0.990 (0.721–1.357)	0.948		
AFP (ng/mL)	>5 vs. ≤5	1.723 (1.234–2.407)	0.001	1.592 (1.135–2.232)	0.007
Liver cirrhosis	Yes vs. No	2.125 (1.616–2.793)	<0.001	2.019 (1.527–2.670)	<0.001
Diabetes	Yes vs. No	2.352 (1.784–3.101)	<0.001	2.214 (1.675–2.927)	<0.001
Child–Pugh grade	B vs. A	2.677 (1.667–4.298)	<0.001	2.310 (1.431–3.731)	0.001
BCLC stage	A vs. 0	1.551 (1.047–2.297)	0.028		
Tumor number	Multiple vs. Single	1.788 (1.101–2.902)	0.019	1.679 (1.029–2.740)	0.038
Tumor size (cm)	>2 vs. ≤2	1.402 (1.024–1.919)	0.035	1.523 (1.109–2.094)	0.009
Histological grade	Poor vs. well + moderate	2.318 (1.188–4.522)	0.014		
Vascular invasion	Yes vs. No	1.639 (1.236–2.173)	0.001	1.498 (1.129–1.986)	0.005
Cholecystectomy	Yes vs. No	0.927 (0.710–1.210)	0.577		
Surgery method	Laparoscopic vs. Open surgery	0.833 (0.504–1.375)	0.474		
Resection type	Lobectomy vs. Segmentectomy	0.876 (0.608–1.262)	0.479		

## Data Availability

The original data are available upon reasonable request to the corresponding author.
